# Draft Genome Sequence of an Extensively Drug-Resistant Mycobacterium tuberculosis Manu-Ancestor Spoligo-International Type 523 Isolate from Thailand

**DOI:** 10.1128/genomeA.01589-14

**Published:** 2015-02-19

**Authors:** Sanjib M. Regmi, Angkana Chaiprasert, Olabisi O. Coker, Areeya Disratthakit, Therdsak Prammananan, Prapat Suriyaphol, Teo Yik-Ying, Ong Twee Hee

**Affiliations:** a Department of Microbiology, Faculty of Medicine Siriraj Hospital, Mahidol University, Bangkok, Thailand; b National Center for Genetic Engineering and Biotechnology, Pathumthani, Thailand; c Bioinformatics and Data Management for Research Unit, Office for Research and Development, Faculty of Medicine Siriraj Hospital, Mahidol University, Bangkok, Thailand; d Saw Swee Hock School of Public Health, National University of Singapore, Singapore

## Abstract

We present the draft genome sequence of DS-16780, with a rare spoligo-international type (SIT) 523 (777777777777771) genotype, which reveals an extensively drug-resistant Mycobacterium tuberculosis (XDR-TB) phenotype. The isolate is a representative of clonal XDR-TB from the western part of Thailand.

## GENOME ANNOUNCEMENT

The emergence and spread of multidrug-resistant (MDR) and extensively drug-resistant (XDR) Mycobacterium tuberculosis pose a threat to global tuberculosis (TB) control (1). In 2013, 100 countries reported XDR-TB cases to the WHO and an estimated 9.0% of MDR-TB cases were XDR-TB (2). XDR-TB has been documented in Thailand since 1997 (3).

Among the XDR-TB isolates collected from 2004 to 2012, we identified a clone with spoligo-international type 523 (SIT523) genotype. We announce here the draft genome sequence of DS-16780, an XDR-TB strain, of the same clone. The strain was obtained from a sputum specimen of a pulmonary tuberculosis patient from Kanchanaburi province, Thailand in 2007. Strains with such a spoligotype pattern were reported from Thailand in 2000 (4). The strains were previously designated “unknown” in the SpolDB4 database (5). With a recent update in SITVITWEB, the strains are reclassified as SIT523, Manu-ancestor and have been reported in 15 countries (4).

Genomic DNA was extracted by the cetyl-trimethyl-ammoniumbromide (CTAB) method and sequenced on an Illumina MiSeq platform. The output yielded an average read length of 250 bp, generating 1,096,288 paired-end reads. The reads were aligned to the M. tuberculosis H37Rv reference (GenBank accession no. NC_000962.3) with Bowtie 2 (version 2.1.0) and analyzed by BEDTools (version 17.2.0). The results showed that 97.45% of the reads were aligned with 98.1% of the reference genome coverage by at least one read.

The reads were assembled by CLC GenomicsWorkbench (version 7.5.1) (http://www.clcbio.com). A draft assembly of 4,240,703 bp with 243 contigs was produced. The contigs have an average length of 17,451 bp, *N*_50_ of 53,646, and an average coverage of 74×. The G+C content of the genome is 65.4%. Annotation was performed by the NCBI Prokarytoic Genome Annotation Pipeline (http://www.ncbi.nlm.nih.gov/genome/annotation_prok/). The pipeline predicted 4,135 genes, 4,010 coding sequences, 44 tRNAs, and 3 rRNAs.

Lineage determination by TB profiler (http://tbdr.lshtm.ac.uk/) identified the isolate as lineage 2.1 (East Asian, non-Beijing) with RD 105 deletion. Single-nucleotide polymorphism (SNP) determination was performed using SNPsfinder (http://snpsfinder.lanl.gov/). Compared to the H37Rv genome, the DS-16780 genome has 1,438 SNPs of which 148 were intergenic, and 1,290 (513 synonymous and 777 nonsynonymous) were found in protein coding regions. Compared to the genomes of other M. tuberculosis strains in NCBI GenBank, DS-16780 has 527, 825, 527, and 471 common SNPs with the genomes of strains EAI5 (NC_021740.1, lineage 1), CCDC5079 (NC_017523.1, lineage 2, Beijing family), CAS/NITR204 (NC_021193.1, lineage 3), and Haarlem (NC_022350.1, lineage 4), respectively, suggesting a close relationship with the Beijing family. A previous whole-genome sequence (WGS) analysis of Percy209, a SIT523 strain, also resulted in clustering of the strain with the Beijing family (6).

SNPs known to confer resistance to rifampin (S450L in *rpoB*), isoniazid (S315T in *katG*), ethambutol (M306I in *embB*), pyrazinamide (I90S in *pncA*), fluoroquinolones (D94G in *gyrA*), and aminoglycosides (a1401g in *rrs)* were observed and confirmed the drug-resistant phenotypes.

The availability of the draft genome of DS-16780 might provide a better understanding of the genetic characteristics of this rare genotype.

### Nucleotide sequence accession numbers.

This whole-genome shotgun project has been deposited at DDBJ/EMBL/GenBank under the accession no. JWIA00000000. The version described in this paper is version JWIA01000000.
